# Leakage does not fully offset soy supply-chain efforts to reduce deforestation in Brazil

**DOI:** 10.1038/s41467-022-33213-z

**Published:** 2022-09-17

**Authors:** Nelson Villoria, Rachael Garrett, Florian Gollnow, Kimberly Carlson

**Affiliations:** 1 Department of Agricultural Economics, Kansas State University, Manhattan, KS 66506 USA; 2 Environmental Policy Lab, ETH Zürich, Zürich, 8092 Switzerland; 3 Department of Geography and Cambridge Conservation Initiative, Cambridge University, Cambridge, UK; 4 Department of Earth and Environment, Boston University, Boston, MA 02215 USA; 5 Department of Environmental Studies, New York University, New York, NY 10003 USA; 6 Department of Natural Resources and Environmental Management, University of Hawaiʻi, Honolulu, HI 96822 USA

**Keywords:** Forestry, Economics, Agriculture

## Abstract

Zero-deforestation supply chain policies that leverage the market power of commodity buyers to change agricultural producer behavior can reduce forest clearing in regions with rapid commodity expansion and weak forest governance. Yet leakage—when deforestation is pushed to other regions—may dilute the global effectiveness of regionally successful policies. Here we show that domestic leakage offsets 43-50% of the avoided deforestation induced by existing and proposed zero-deforestation supply chain policies in Brazil’s soy sector. However, cross-border leakage is insignificant (<3%) because soybean production is displaced to existing U.S. farmland. Eliminating deforestation from the supply chains of all firms exporting Brazilian soy to the EU or China from 2011-2016 could have reduced net global deforestation by 2% and Brazilian deforestation by 9%. Thus, if major tropical commodity importers (e.g., the EU) require traders to eliminate deforestation from their supply chains, it could help bend the curve on global forest loss.

## Abstract (web summary)

This research quantifies the role of zero deforestation policies and potential leakages in Brazilian soybean production, the third major driver of deforestation globally. Here the authors provide the first estimates of net global avoided soy-driven deforestation from zero-deforestation import restrictions and find that such restrictions could help avoid ~40% of deforestation for soy cultivation in Brazil and ~2% of global deforestation.

## Introduction

Zero-deforestation supply chain policies leverage the influence of downstream companies to enforce stringent sourcing requirements from upstream farmers. The commitment by private actors to adopt more sustainable producing practices may help to overcome the limited political will and capacity for deforestation control in producing regions. Starting in the mid-2000s, under pressure from non-governmental environmental organizations^1^, a handful of the world’s largest agricultural trading companies voluntarily committed to eliminate deforestation from their supply chains^2^. For instance, signatories to the Amazon Soy Moratorium (ASM) in Brazil—the first implemented voluntary set of zero-deforestation commitments—only buy soybeans produced in areas where forests were cleared long ago^3^. Since then, hundreds more companies in the agri-food and timber sectors have pledged to address deforestation associated with the products they handle^1^.

Supply chain approaches to forest conservation are also being considered by concerned importing governments. The European Union (EU) is proposing legislation to limit imports of soybeans and other forest-risk commodities to importing companies that can verify the deforestation-free status of their products^4,5^. Similar legislation is being considered in the United States (US) and the United Kingdom^6–9^.

Eliminating deforestation embodied in supply chains starts in production regions by ensuring that no forested lands are converted to the commodity in question. Whether such land use restrictions adequately address global forest loss depends on the scale and location of their adoption^1,10^ as well as the degree to which deforestation reductions are offset by the displacement or “leakage” of forest loss to other regions^11,12^. Restricting forest conversion to agriculture in the target region can lead to deforestation leakage when it changes commodity prices, land prices, and the relative competitiveness of different regions^13–15^. Such leakage is likely to occur whenever the geographic scope of an intervention is limited relative to the overall scope of the targeted activity^10^. Therefore, leakage is potentially a substantial barrier to the effectiveness of zero-deforestation supply chain policies because land conversion restrictions apply only to a fraction of total production^2^.

Yet, identifying deforestation leakage, which can span continental to global scales, remains a major empirical challenge. Simultaneous changes in markets, governance, and climate can confound attempts to attribute changes in the potential spillover regions to specific policy changes in the target region^16,17^. Previous impact assessments of voluntary zero-deforestation commitments focused only on past or potential future leakage from the ASM to other regions in the Amazon^17–19^ or within the Cerrado^20^. Moreover, many larger scale zero-deforestation supply chain policies (e.g., companies’ current global zero-deforestation pledges) and all proposed zero-deforestation import regulations have not yet been implemented, preventing retrospective (ex-post) statistical analysis of these policies.

Our research advances the study of zero-deforestation supply chain policies by increasing the scope over which leakage is assessed and using scenario modeling to examine both existing historical policies and potential alternatives. We assess the degree to which leakage offsets reductions in deforestation and associated greenhouse gas (GHG) emissions achieved by the implementation of zero-deforestation policies in the Brazilian soybean supply chain. In contrast to previous assessments, we evaluate leakage across all the producing regions in Brazil and to other countries. In addition to voluntary zero-deforestation commitments we include scenarios of zero-deforestation supply chain policies driven by import regulations in the EU and China that are not yet implemented but that play an important role in current discussions about the future of supply chain interventions in strengthening forest conservation in Brazil^4,20,21^.

We estimate how much avoided deforestation in Brazil from 2011–2016 is displaced to neighboring agricultural forest frontiers in Bolivia, Argentina, and Paraguay (“BAP”), land-abundant countries with carbon-rich forested ecosystems and ample scope for further expansion of agriculture^22^, and to other oil crop producing regions. We compare five policy scenarios: (1) the ASM; (2) the ASM plus pledged, but yet to be implemented, global voluntary zero-deforestation commitments in Brazil’s soy supply chain^23^; (3) adoption of zero-deforestation supply chain policies by all companies that export to the EU as may be required under legislation under consideration by the EU^5^; (4) adoption of zero-deforestation supply chain policies by all companies that export to China, the leading soybean importing nation^24^ whose most important transnational soy trader has signaled possible adoption of zero-deforestation supply chain policies for their imports^21^; and (5) adoption of zero-deforestation supply chain policies by companies that export to either China or the EU. Supply chain differentiation within the same company requires monitoring, tracing, and certification systems with separate chains of custody. We assume this is more costly than transforming the entire supply chain, as demonstrated by difficulties in separating transgenic and conventional soy and reliance on mass-balance approaches for certified products^25^. Thus, for scenarios three to five we assume that companies that sell to the regulated markets transform their entire supply chain to be deforestation-free.

Traders with more market share likely have greater leverage over producer behavior. The minimum regional market coverage by committed traders needed to achieve regional zero-deforestation crop production—defined here as cultivation of soybeans or other crops only within areas deforested before 2011 (Methods)—remains uncertain^1^. We thus examine three thresholds for compliance and report the ≥75% market coverage threshold in the main text; all thresholds result in global net avoided deforestation. We circumvent the empirical measurement challenges highlighted above by applying the GTAP-AEZ model and database^26^ with Brazil’s agro-ecological zones remapped into Brazilian biomes. The GTAP-AEZ modeling framework has been used to analyze deforestation leakage associated with biofuel mandates^27,28^, policies to reduce deforestation in the oil palm sector^15^, and the tariffs levied by China on US imports, including soybeans, meant to retaliate against US trade sanctions^29,30^. Like the import regulations studied here, all of these policies leverage market forces to influence the land use decisions of agricultural producers, and thus have the potential to displace production across countries.

**Fig. 1:**
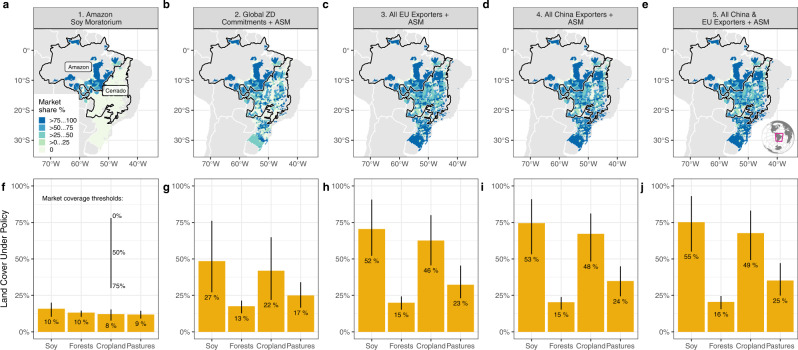
Spatial footprint of zero-deforestation supply chain policy scenarios. **a**, **b** Municipality-level market shares of companies with voluntary zero-deforestation commitments [Amazon Soy Moratorium (ASM) and Global ZDCs]. **c**–**e** Municipality market shares of companies that export to the EU (**c**), China (**d**), or both (**e**). Panels (**f**–**j**) depict the percentages of Brazil’s soy and land covers subject to these zero-deforestation supply chain policy, with vertical lines providing the range of outcomes based on >0% [top of line], ≥50% [intersection of line with bar] and ≥75% [bottom of line] thresholds used to determine if a municipality is subject to zero-deforestation supply chain policies. The number (%) indicates coverage under the most restrictive market coverage threshold (≥75%). For instance, in the ASM scenario, 10% of Brazil’s forests (located in the municipalities with a ≥75% market share in panel (**a**)), cannot be converted to agriculture. Thus, increases in soy area would occur by converting non soy cropland or pastures within Brazil, or elsewhere in the world. Sources: TRASE^50^ and Mapbiomas^54^.

We determine the shares of each biome in Brazil under zero-deforestation supply chain policies using recently available market data (Fig. 1, Methods,). This strategy allows us to track changes in agricultural land use and GHG emissions within and across biomes and across countries. We present our results as the difference in deforested area and GHG emissions between baseline (Supplementary Information S1) and policy scenarios. Our simulated baseline accurately reproduces the exports of soybeans to China by Brazil and to a lesser extent, by Bolivia, Argentina, and Paraguay, which in contrast to Brazil tend to have more variable exports (Supplementary Information S1). It also accurately tracks the direction of observed change in oilseed, cropland, and forest area in the BBAP countries, although tends to underestimate changes in areas. This is due both to differences in definitions of land covers between the GTAP database and FAOSTAT datasets (Supplementary Information S1) and to the importance of localized processes (e.g., fires, land property rights, local ordinances) on land use dynamics that are difficult to capture using a multi-country, multi-commodity model.

Here we show that due to the market structure of global soy markets, there is ample scope to eliminate soy-driven deforestation by extending zero-deforestation supply chain policies within Brazil without significantly threatening forests elsewhere or disrupting international markets. Extensive systematic sensitivity analysis shows that results are robust to uncertainty in the key parameters regulating land use in the GTAP-AEZ model (Supplementary Information S2).

## Results

### Half of Amazon Soy Moratorium impact absorbed by leakage

**Fig. 2:**
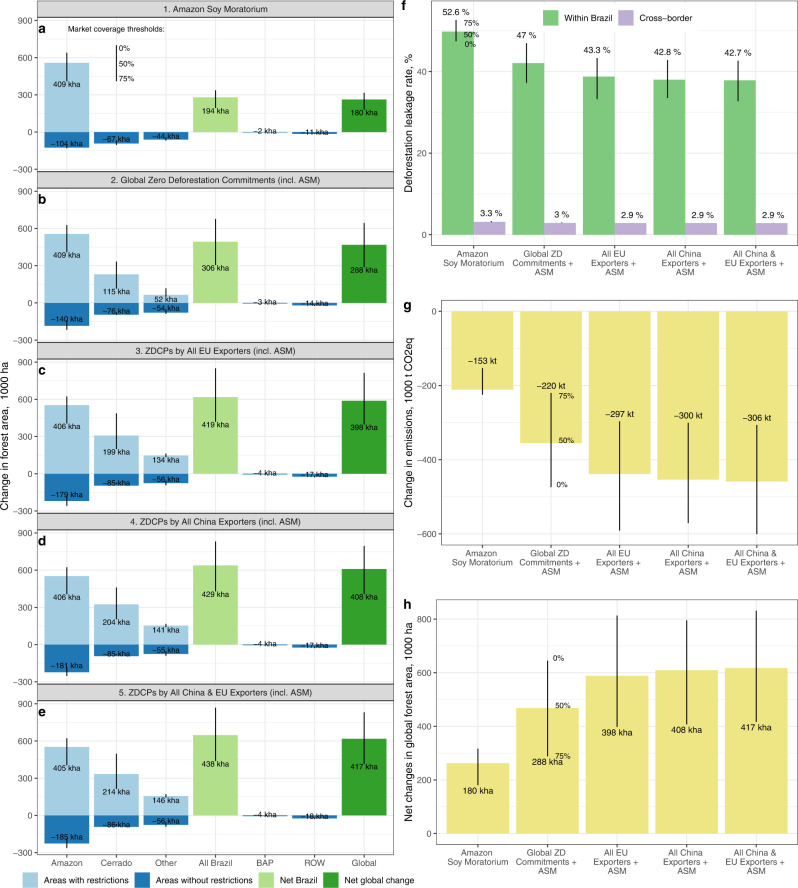
Changes in forest area and greenhouse gas emissions relative to a baseline without land use restrictions across scenarios and regions. **a**–**e** Changes in forest area. Positive values in indicate gross avoided deforestation while negative values are gross displaced deforestation. **f** Deforestation leakage rates defined as the ratios of displaced deforestation within Brazil and to the rest of the world as percentages of the gross avoided deforestation from zero-deforestation supply chain policies (Methods). **g** Net changes in greenhouse gas emissions from land use change (Methods). **h** Net changes in global forest area. In all cases the height of the bars corresponds to the difference between counterfactual and baseline values obtained under the market share threshold (≥50%). Vertical lines indicate the range of outcomes based on >0% [top of line], ≥50% [intersection of line with bar] and ≥75% [bottom of line] thresholds used to determine if a municipality is subject to zero-deforestation supply chain policies. The displayed values in all the plots are for the most restrictive scenario (≥75% market share). BAP = Bolivia, Argentina, Paraguay; ROW = rest of the world.

We find that the ASM led to 409 kha of gross avoided deforestation within the Brazilian Amazon from 2011 to 2016 (82 kha/year; Fig. 2a). This avoided forest loss rate is similar to Heilmayr et al.’s^17^ lower bound estimate of ~90 kha/year (Supplementary Information S3). Around half of this avoided deforestation was offset by increases in deforestation in parts of the Amazon outside the ASM, generating a within-Brazil leakage rate of 53% (Fig. 2f). Domestic leakage was high because only 10% of Brazil’s forests were in municipalities where committed soy company market share exceeded 75% (Fig. 1f).

After accounting for leakage within Brazil, net avoided deforestation in the Amazon and Cerrado biomes totaled 238 kha, about 23% of the 847 kha deforested for soy cultivation in both biomes during the same period (Supplementary Information Fig. S1). Net avoided deforestation within Brazil amounted to 194 kha (Fig. 2a), or 4% of total observed forest loss in the country^31^ (Supplementary Information Fig. S4). Cross-border deforestation spillovers to the BAP region were negligible (<2 kha increase), and deforestation in the rest of the world increased by 11 kha, for a cross-border leakage rate just above 3% (Fig. 2f). Global net avoided deforestation in this scenario totaled 180 kha (Fig. 1a), 0.9% of global deforestation during 2011–2016^31^, and reduced GHG emissions by 153 kilotonnes CO_2_ equivalent (kt CO_2_e; Fig. 2g), approximately 0.004% and 0.01% of global (4018 megatonnes [Mt] CO_2_e) and Brazil’s (1730 Mt CO_2_e) GHG emissions due to land use change and forestry (LUCF) during the same period, respectively^32^.

### Global voluntary zero-deforestation commitment implementation would help protect the Cerrado biome

Implementation of all global voluntary zero-deforestation commitments in Brazil’s soy supply chain, including the ASM, across Brazil from 2011–2016 would have increased gross avoided deforestation by 167 kha (Fig. 2b) and generated Brazil-wide gross forest savings 40% higher relative to the ASM-only scenario. Combined net avoided deforestation in the Amazon and the Cerrado represents 36% of the 847 kha of observed deforestation for soy in those biomes (Supplementary Information Fig. S1). Our finding of 39 kha (~8 kha/year) of net avoided deforestation in the Cerrado (Fig. 2b) is considerably lower than the projections by Soterroni et al.^20^ of ~120 kha/year from 2020–2050. The difference is partly explained by our use of market coverage thresholds to isolate areas most likely to be impacted by zero-deforestation supply chain policies, whereas they assume a complete and uniform application of commitments across the biome, irrespective of the sourcing locations of companies with zero-deforestation commitments.

The leakage rate in this scenario (47%, Fig. 2f) is similar to leakage in the ASM-only scenario because the addition of global commitments only increases the proportion of Brazil’s forest area subject to land use restriction from 10% to 13% (Fig. 1f, g). Net avoided deforestation in Brazil would have amounted to 306 kha, almost twice as much as in the ASM scenario and 6.2% of the total deforestation experienced by Brazil during 2011-2016 (Supplementary Information Fig. S4). Spillovers into the BAP region and the rest of the world were about 3 kha and 14 kha, respectively, for a cross-border combined leakage rate of 3% (Fig. 2f). Net global deforestation in this scenario amounted to 288 kha, 1.4% of global deforestation during the period, and avoided GHG emissions totaled 220 kt CO_2_e (Fig. 2g), 0.005% and 0.01% of global and Brazil’s LUCF GHG emissions, respectively.

### Minimal regional deforestation leakage explained by destination market segmentation

**Fig. 3:**
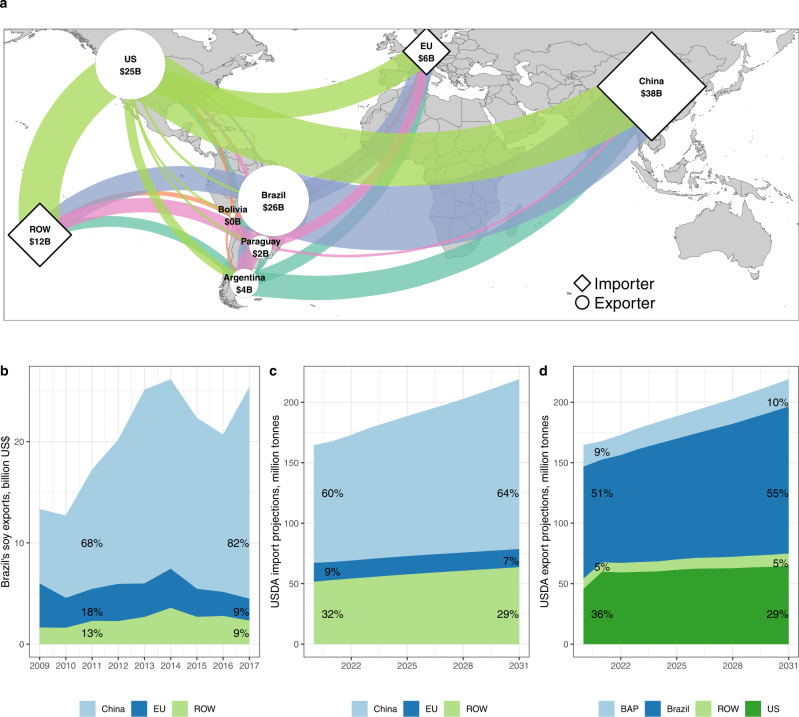
Illustration of China’s increasing dominance in global soy markets. **a** Main international soy trade flows (billion US$; average 2017–2018) indicate that world trade is highly concentrated with China importing 68% of global exports, 93% of which comes from the US and Brazil. **b** The main destinations of Brazil’s soy exports (2009–2017) indicate that China’s importance in Brazil’s soy exports has grown rapidly, from 68% in 2011 to 82% in 2017, while the EU’s share of Brazil’s soy exports has decreased, from 18% to 9%. **c**, **d** Projected sources of future long-term (10-year) soy import demand (**c**) and export supply (**d**) indicate strengthening demand in China, which will be mostly satisfied by Brazil. Sources: **a**–**b**^63^, **c**–**d**^64^. BAP = Bolivia, Argentina, Paraguay; ROW = rest of the world.

The low rates of deforestation leakage to the BAP region are explained by the modest effect of zero-deforestation supply chain policies on global oil crop production and limited price transmission within the BBAP (Brazil + BAP) region (Supplementary Information S1). Intra-BBAP soybean trade volume was relatively small from 2011–2016 and remains this way (Fig. 3a). In addition, China is the main market where US and Brazil producers compete, while Bolivia and Paraguay predominately supply EU markets (Fig. 3a). This pattern of destination-market segmentation partly disconnects BAP and Brazil producers and tightly connects the supply responses of farmers in the US and Brazil^24,33^ so that reduced soybean production in Brazil due to deforestation restrictions is largely absorbed by increases in US soybean area into existing farmland (Supplementary Information S4). Such absorption occurs despite increasing demand for ethanol within the United States over the 2011–2016 period (Text S1). Although additional conversion of forested lands in the US is minimal (<150 ha, Fig. S9), negative spillovers to non-forest ecosystems in the US, such as prairies and wetlands in the US, may still occur. A consequence of this market segmentation is that the low cross-border leakage associated with soy zero-deforestation supply chain policies may not apply to other deforestation-risk commodities. For instance, oil palm is largely restricted to climates that also support humid tropical forests and cross-border leakage rates are substantially higher that what we found for Brazil^15^.

### Importer regulations could substantially increase footprint and impact of supply chain policies

The share of Brazil’s soy area where companies that export to the EU have a market share of ≥75% of soy export volumes is twice the area under voluntary global zero-deforestation supply chain policies (52% vs. 27%, Fig. 1h). Still, only 15% of Brazil’s forests would be subject to land use restrictions under a scenario in which EU regulations had been in place from 2011-2016 (Fig. 1h), generating a domestic leakage rate of 43% (Fig. 2f). In this scenario, net avoided deforestation in Brazil would have amounted to 419 kha (Fig. 2c), 8.5% of Brazil’s observed deforestation during the period. This is a 37% increase in forest savings relative to the adoption of voluntary global zero-deforestation commitments. Net forest loss in the Amazon and the Cerrado would have totaled 340 kha, less than half (40%) of the observed deforestation for soy cultivation during 2011–2016 in these biomes (Supplementary Information Fig. S1).

Despite greater coverage of zero-deforestation supply chain policies under this scenario (Fig. 1c, h), cross-border international leakage remains close to 3% (Fig. 2f) due to the destination-market segmentation discussed above. This scenario also results in 35% less GHG emissions relative to the voluntary global zero-deforestation commitments scenario (−297 kt CO_2_e, Fig. 2g), or 0.007% and 0.02% of global and Brazil’s LUCF emissions, respectively. Worldwide net forest savings amount to 398 kha (Fig. 2c), or 1.87% of global deforestation, and a 38% increase in global net avoided deforestation relative to the adoption of global voluntary zero-deforestation commitments.

The EU Commission estimates that mandatory supply chain due diligence and certification aimed at stopping deforestation driven by EU demand for six forest-risk commodities including soy^5^ will avoid the deforestation of 72 kha/year starting in 2030. This estimate is far more conservative than our result of 80 kha/year considering that it covers more commodities and countries.

If the soy trading companies that export to China had imposed zero-deforestation requirements from 2011–2016, avoided deforestation and GHG emissions (429 kha of net avoided deforestation in Brazil, 408 kha net global forest savings, −300 kt CO_2_e emissions, Fig. 2d, 2g) would have been similar to those stemming from EU-mandated commitments. This similarity is driven by the fact that most companies in Brazil sell to both China and the EU and is confirmed by the similarity of results from the final scenario. We find little difference in forest conservation whether zero-deforestation requirements cover all exporters from Brazil to both China and the EU (scenario 5, Fig. 2e), only exporters to the EU (scenario 3, Fig. 2c), or only exporters to China (scenario 4; Fig. 2d).

### Import regulations can help avoid more deforestation globally but come with risks

Taking the ratio of domestic to cross-border leakage as an indicator of the effectiveness of zero-deforestation supply chain policies, our results suggest that supply chain efforts to halt soy-driven deforestation would be substantially more effective if extended to the rest of Brazil, especially if they include zero-deforestation requirements for exporting to the EU and/or China. Our key assumption is that such regulations incentivize traders to implement zero-deforestation policies (e.g., by strengthening monitoring systems and engaging with governments to reduce commodity-driven deforestation) across all existing and future sourcing regions rather than segregating their supply streams or leaving such demanding markets. If this assumption holds true, the current EU proposal to halt import-driven deforestation could trigger widespread structural change in the implementation of deforestation control efforts.

Scaling up zero-deforestation regulations for Brazil’s soy entering the EU is not without risks. While China’s share of Brazil’s total soybean exports grew from 68% in 2011 to 82% in 2017, the EU’s share declined from 18% to 9%, reducing its overall market power (Fig. 3b). In the next decade most growth in soybean demand will come from China (Fig. 3c), which is projected to be satisfied almost entirely with additional exports from Brazil (Fig. 3d). The dependency of Brazil’s soybeans on China’s market may be exacerbated by the onset of the US-China trade war in early 2018, which could trigger significant increases in deforestation^30^. Traders may choose to drop out of the EU markets or segregate their supply chains into compliant and non-compliant streams if the benefits of selling to regulated EU markets exceed the costs of implementing zero-deforestation supply chain policies across Brazil. This would reduce gross avoided deforestation relative to our analysis and could also weaken the influence of EU-based traders on state-led forest governance in Brazil. Stringent zero-deforestation supply chain approaches may substantially impact rural livelihoods in deforestation risk areas^34^ if excluding non-compliant actors is cheaper than engaging with them. Moreover, a focus on protecting forests may generate spillovers to non-forest ecosystems. Building capacity among producers (via financing and training) to address non-compliance and improve existing agricultural practices, coupled with reducing incentives for firms to avoid non-compliant actors, may help reduce such negative spillovers from zero-deforestation supply chain policies^34^.

## Methods

### Modeling framework

We used an open source, fully documented, and publicly available medium run applied general equilibrium (AGE) model^35^ with explicit treatment of subnational land markets divided in Agroecological Zones (AEZ), nicknamed GTAP-AEZ^26^. The GTAP-AEZ framework is based on decision nests at which agricultural producers decide on land cover conversions (Supplementary Information Fig. S2), for example, from pastures to cropland, and then on the allocation of individual crops within the cropland. As producers in different AEZs are connected through land, labor, and capital markets, competition among land uses, and supply chains, the GTAP-AEZ model is ideally suited to study within-country changes in land use across AEZs. Moreover, through an explicit treatment of international trade flows, the GTAP-AEZ framework allows for tracking the effects of regional policies on land use patterns in other countries.

We updated the standard GTAP-AEZ model to include a nesting structure that separates the decision to convert forest to agricultural land from the decision to convert pasture to cropland, which is justified by the observation that deforested lands transition first into pastures, and then onto cropland^36^. This nesting structure applies to all the regions. We also adopted regional elasticities of transformation, from natural covers to agricultural land, and between pastures and cropland, calibrated based on recent historical changes^36^. We further updated the income elasticities of demand for agricultural and food products to reflect the latest work in this area^37^. A critical assumption underlying the GTAP-AEZ framework is the productivity of marginal, hitherto, uncultivated lands, as it determines the extensive margin of land expansion. Another key assumption in the GTAP-AEZ model is the response of yields to changes in commodity and input prices^38^. For both we use the assumptions in the original GTAP-AEZ model^26^. Given the uncertainty regarding these parameters, we conduct extensive systematic sensitivity analysis of our results to alternative parametric configurations (Supplementary Information S2).

Underlying the model there is a database that consistently represents production, consumption, and trade patterns of 140 regions and 57 sectors in year 2011^39^. To make solution times and model output manageable, we aggregated the model into 11 regions: Brazil, Bolivia, Argentina, Paraguay, Rest of Latin America, US-Canada (North America), European Union (28 countries), China, Malaysia and Indonesia, sub-Saharan Africa, and the rest of the world. We also collapsed the 57 commodity sectors into 18 sectors (i.e., paddy rice, wheat, coarse grains, oilseeds, raw sugar, grazing livestock, non-grazing livestock, forestry, extractive industries, processed livestock, vegetable oils, processed rice, processed sugar, other processed food, chemicals, manufactures and services). For Brazil, we considered the GTAP aggregate oilseed commodity as soybeans because soybeans account for more than 96% of oilseed production in Brazil^40^. The database is complemented with data on agricultural land rents by land use and natural land covers at the level of Agroecological Zones (AEZ), also representative of 2011^41^.

### Spatial footprint scenarios (SFS) and market share thresholds of zero-deforestation supply chain policies

The SFS (Fig. 1) are designed to assess how much deforestation would be avoided by implementing different configurations of the company- and importing country-led zero-deforestation supply chain policies. Except for the ASM, most policies–either voluntary or imposed—have not been implemented^1,4,7,21^. Therefore, the SFS are counterfactual, non-observed states of the world. We estimate changes in deforestation and other economic outcomes as the difference between the counterfactual SFS and a baseline (as explained below). The baseline includes patterns of land use, land cover, and other economic outcomes obtained by letting the model simulate the changes in equilibrium as the economy responds to a set of drivers of land use during the period 2011-2016 (GTAP Database and AEZ Database, V9^39,41^), without any land restriction to land expansion in Brazil.

The economic drivers include macroeconomic indicators (Supplementary Information Table S1), changes in agricultural factor and input productivity (Supplementary Information Table S2), and demand for biofuels (text S1). For each of these indicators, the value in 2011 is the average from 2010–2012 and the value in 2016 is the average from 2015–2017. These three-year periods are intended to smooth annual fluctuations in the different indicators. We use data up to 2017 in our analysis given the significant turbulence in soybean and other agricultural markets brough about by the US-China trade war started in January 2018. Soybeans, central to our analysis, saw a large divergence in the export prices to China charged by the US and Brazil^42^. Although the price gap eventually closed, the direction of trade flows changed significantly, especially for the U.S^43^. Such turbulence was exacerbated by the global efforts to contain the spread of COVID-19, which triggered policy responses with potential significant worldwide effects on food consumption, production, and distribution^44^. The export market shares of all the companies active in the Brazil’s soybean market from the Trase v2.4 database, are also available up to 2017.

The results over a five-year term horizon are representative of an economic medium run^45^, which is long enough to allow economic agents to adjust their production and consumption patterns to the changes in prices brought about by land use restrictions. By focusing on a medium run we avoid the rigidities of economic short-run assumptions (i.e., lack of supply and demand response) as well as the significant uncertainties of economic analysis in the long-run (i.e., uncertain, or unpredictable future economic growth trajectories, technologies, and changes in international trade patterns).

The ASM was in place during our period of study, and it should be considered a baseline relative to further hypothetical policy developments analyzed here. The drawback of including the ASM in the baseline is that we would not be able to report the ASM outcomes, which, by virtue of its pioneering status, is a natural benchmark of future zero-deforestation policies in Brazil’s soybean supply chain. For this reason, we exclude the ASM from the baseline by not imposing land restrictions in the Amazon.

The SFS we evaluate are as follows:

1. Amazon Soy Moratorium. This scenario uses the spatial footprint in the Brazilian Amazon of the companies that implemented the moratorium in 2006. These companies are: Abc Industria, ADM, Amaggi, Bunge, Cargill, Louis Dreyfus, Seara, Fiagril, Nidera, Noble, Cofco, Baldo, Imcopa, Agrex, CHS, Coamo, Engelhart CTP, Gavilon, Glencore, Invivo, Marubeni, Multigrain, Nova Agri, Olam, Perdue, Sodrugestvo, Timbro, and Selecta. Other companies that are part of the ASM do not export soybeans from the Amazon are: Binatural, JBS, Oleos menu, Agribrasil, and Culturale. The duration of the ASM has been extended indefinitely^17^.
2. Global voluntary Zero-deforestation Commitments + Soy Moratorium. This scenario includes the ASM companies above and adds all global voluntary zero-deforestation commitment as if they were implemented in 2011. The global commitments have been pledged by a subset of the companies that agreed on the ASM. These are (pledge year and in parentheses): ADM (2015 company pledges); Amaggi (2017 company pledges), Bunge (2015 company pledges) Cargill (2014 New York Declaration on Forests); Louis Dreyfus (2018 company pledges); Cofco (2019 Soft Commodities Forum); Glencore (2019 Soft Commodities Forum); and Denofa do Brazil (2014 New York Deforestation of Forests).
3. Import restrictions imposed by the European Union (EU). Agriculture-driven deforestation has become an increasingly polarizing issue between the EU and Brazil, and is a central issue in a potential trade agreement between the EU and the MERCOSUR, a trade bloc agreement among Argentina, Brazil, Paraguay, and Uruguay^46^. We therefore also explore the effects of the adoption by the EU of mandatory rules currently considered by the European Parliament that would de facto require the implementation of zero-deforestation supply chain policies by the companies sourcing soybeans from Brazil^4^. We consider 155 traders exporting to EU plus Switzerland and the United Kingdom that would only procure their soybeans from areas already converted to agriculture prior to 2011. The EU countries appearing as importers consist of Belgium, Bulgaria, Croatia, Cyprus, Denmark, Finland, France, Germany, Greece, Hungary, Ireland, Italy, Latvia, Lithuania, Netherlands, Poland, Portugal, Romania, Slovakia, Slovenia, Spain, and Sweden.
4. Hypothetical import restrictions imposed by China, assumed to be similar to those being currently evaluated by the EU^21^. We consider 140 traders that export to China including Hong Kong (in addition to the ASM) that would only procure their soybeans from areas already converted to agriculture prior to 2011.
5. Hypothetical import restrictions imposed by both China and the EU. These simulations provide an upper bound estimate of the ZCDPs.

In the SFS 3-5 (scenarios 3–5 in the main text) we assume that if a company supplying either EU or Chinese markets decides to produce deforestation-free soybean to preserve market share in one destination, the company will apply those restrictions to their entire supply chain. In other words, we do not allow for different supply chains from the same trader when exporting to different destinations. This is a realistic assumption as companies consider supply chain differentiation very costly due to the unwieldy procedures that would be needed to monitor, trace, and certify production^25,47^.

### Market share thresholds

Uncertainties exist regarding the critical market share (i.e., the percentage of total regional market share held by corporations with zero-deforestation supply chain policies) needed to discourage farmers from selling soybeans produced in recently cleared land to non-committed traders^1^. If no soybean buyers within a region have a zero-deforestation supply chain policy, farmers have no incentives to avoid forest for soybean clearing. Alternatively, if only committed traders buy soybeans within a region, producers should be forced to comply with the zero-deforestation land use restrictions to sell their soybeans. In many Brazilian regions, traders with and without zero-deforestation supply chain policies purchase soybeans, thus producers with soybeans that are not zero-deforestation can typically sell their products. We posit that with increasing regional zero-deforestation supply chain policies market share, the difficulty of selling non-compliant soybeans increases. At some critical market share threshold, farmers may be completely disincentivized from producing soybeans on non-compliant lands that were recently deforested due to the difficulties in selling their product^48,49^.

We bound the uncertainty about the competition structure needed to ensure compliance through three market share thresholds built using the export market shares of all the companies active in the Brazil’s soybean market from the Trase v2.4 database^50^:

1. The most restrictive market share thresholds requires that at least 75% of the soybeans exported from a given municipality are bought by companies with voluntary zero-deforestation commitments. In this scenario, 10% of the area under soybeans in Brazil is subject to the ASM (Fig. 1f). By adding pledged global voluntary zero-deforestation commitments in other biomes to the ASM, 27% of Brazil’s soybean area would be under agreements to halt forest conversion for soy production (Fig. 1g).
2. A less conservative market share thresholds requires an export threshold of 50%. Under this scenario, the area under soybeans that is affected by global voluntary zero-deforestation commitments under the current pledges amount to 48% of Brazil’s total soybean area (Fig. 1g).
3. The least restrictive scenario requires at least one committed company to be present in the municipality (>0% of market share covered by voluntary zero-deforestation commitment). Under global voluntary zero-deforestation commitments, 75% of Brazil’s soybean area would be subject to forest conversion restrictions (Fig. 1g).

### Land cover definitions

We use two different definitions of forests in Brazil to accommodate different biome characteristics and land restriction targets^51^. Definition A was exclusively based on mapped forest cover. Definition B included natural grasslands outside the Amazon Biome, which may have high conservation value and are included in some traders’ zero-deforestation voluntary commitments [e.g., “Transforms our supply chain to be zero-deforestation while protecting native vegetation beyond forests.”^52^]:

- Forest definition “A”: Forest is defined as forest only, as mapped by PRODES for the Amazon^53^ and by Mapbiomas for other biomes [Mapbiomas v4^54^, classes 1, 2, 3, 4, and 5]. Forest area was derived excluding forest regrowth, with forest base year of 2006^54^. We used PRODES for the Amazon biome, because PRODES deforestation maps define the baseline for ASM monitoring, implementation, and enforcement. We used Mapbiomas outside the Amazon Biome. To our knowledge, Mapbiomas provides the most accurate and consistent large scale land use and land cover classification for Brazil.
- Forest definition “B”: Forest is defined as forest only in the Amazon biome, as mapped by PRODES, and forest and grasslands in all other Brazilian biomes, as mapped by Mapbiomas.

### Land use and land cover databases

In addition to the data on soybean export market share and forests, we gathered municipality-level data on agricultural land cover from Mapbiomas v4^54^: total cropland [classes 18–20] and pasture area [class 15], soybean area (ha), soybean production (tonnes), areas with both maize and soybean (ha), maize second harvest area (ha), calculated as the area of second harvest maize that is greater or equal to the area of soy harvested^55^, and cattle headcount (heads)^56^. These data were used to build the different versions of the GTAP-AEZ database, as explained below.

The supply-side spatial footprint scenarios, market share thresholds, and forest definitions give rise to thirty different databases with land use and land cover in each Brazilian municipality (five SFS * three market share thresholds* two Forest Definitions = thirty databases.) We use these land use/land cover databases to build biome-specific distributions of land cover and soybean production with and without zero-deforestation supply chain policies that can be used to calibrate the counterfactual experiments using the GTAP-AEZ model.

### Model calibration

We use the databases discussed in Supplementary Information S1 to recalibrate the GTAP-AEZ model so that Brazil is split into biomes instead of the standard AEZs. This requires rebuilding the original GTAP and GTAP-AEZ databases. The algorithm to split Brazil’s agricultural output values into biomes proceeds as follows. For each spatial configuration of zero-deforestation policies, market share threshold, and forest definition, we use the following algorithm:

1. Overlay the AEZ map used in the GTAP-AEZ database^41^ on a municipality-level map of Brazil^57^. In case that a municipality is split across more than one AEZ, assign the municipality to the AEZ with the largest intersection.
2. Overlay a biome map over the AEZ and municipality maps for Brazil. The biomes generally encompass several AEZs and the same AEZ can occur in different biomes. Biomes other than the Amazon and Cerrado are in an “Other” category. In case that a municipality is split across more than one biome, we assign the municipality to the biome with the higher policy implementation stringency, prioritizing the Amazon, second the Cerrado, and all other.
3. Each municipality receives a unique id for each biome-AEZ combination, for example: AEZ5 becomes AEZ5-Amazon, AEZ5-Cerrado, and AEZ5-Other.
4. Compute compliant market share thresholds for each municipality (using pledges as of 2017), and then categorize the biome-AEZ ids into compliant and non-compliant based on the SFS. For example: AEZ5 becomes AEZ5-Amazon-ZDC, AEZ5-Cerrado-ZDC, and AEZ5-Other-ZDC, and AEZ5-Amazon-Non-ZDC, AEZ5-Cerrado- Non-ZDC, and AEZ5-Other-Non-ZDC.
5. Use aggregate municipality-level land cover (cropland, pasture, and forest area) and land use (soybean area, soy production, areas with both maize and soy, cattle headcount) to assign land cover and land use areas to each biome-AEZ-market share thresholds level.
6. For all regions other than Brazil, build a conventional GTAP-AEZ database representative of 2011. This step uses a database of land use and land cover areas at the level of AEZs^41,58^ to split the country-level output value of relevant products (crops, grazing livestock, and forestry) in the standard GTAP database^39^ into AEZs.
7. For Brazil, we use the AEZ-Biome area and production shares created in steps 1–4 to split the aggregate output values of oilseeds, coarse grains, grazing livestock, and forestry into biomes. Each new database represents a counterfactual year 2011 in which some of the area in each AEZ-BIOME was under a voluntary zero-deforestation commitment pledged before 2020. The simulations answer the question: how different area, production, and consumption would have been in 2016 if the pledged commitment had been in place since 2011.
8. The area of the crops other than oilseeds and coarse grains (paddy rice, etc.) are shared out in each biome-AEZ in proportion to the cropland.

### Implementation of zero-deforestation supply chain policies and deforestation leakage channels in the GTAP model

In each experiment we halt land conversion between forest and agriculture in the areas assumed under ZDCs by way of a subsidy that compensates producers for the economic losses of not transforming forests on to agriculture. Halting forest conversion in Brazil induces a shortage of agricultural land which drives up land rents in agriculture relative to other land uses. The effects of heightened land scarcity in Brazil may be transmitted to other regions of the world through changes in commodity prices. The strength with which these price changes affect other countries depends on Brazil’s global market shares of the commodity in question, and on the extent of competition in destination markets. In turn, changes in commodity prices alter the relative profitability of alternative land uses in these regions. Either in Brazil or abroad, leakage occurs as the higher returns to agricultural land incentivize land expansion into forests without zero-deforestation policies. Changes in land use are accompanied by a relocation of factors of production (land, labor, and capital) and other inputs (e.g., fertilizer) toward the production of the most profitable commodities. In addition to the reallocation of inputs, the model allows for substitution of non-land inputs for land in response to higher commodity prices.

### Deforestation leakage rates

Following the literature on carbon leakage^59^, we define the deforestation leakage rate as the increase in deforestation in regions without restrictions (*No ZDSP*, where ZDSP stands for zero-deforestation supply chain policy) induced by the measures taken in regions with zero-deforestation policies (*ZDSP*) as a percentage share of the absolute value of deforestation in regions with ZDSP. Formally:

1 $${Deforestation\; Leakage\; Rate}=\frac{\Delta {Deforestatio}{n}_{{No}{ZDSP}}}{{{{{{\rm{|}}}}}}\Delta {Deforestatio}{n}_{{ZDSP}}{{{{{\rm{|}}}}}}}\times 100.$$

Where $\Delta$ denotes the difference between deforestation outcomes in the baseline and counterfactual scenarios, and

2 $$\Delta {Deforestatio}{n}_{{Global}}=\Delta {Deforestatio}{n}_{{ZDSP}}+\Delta {Deforestatio}{n}_{{No}{ZDSP}}.$$

### Greenhouse gas emissions

Greenhouse gas emissions (GHGs) from the changes in land cover associated with the different experiments are calculated using the open-source AEZ Emission Factor (AEZ-EF) Model^60^. The AEZ-EF model closely follows IPCC GHG inventory methods and relies on its default values. The model includes cover-specific (cropland, pastures, and forests) subnational carbon estimates for biomass (above and below-ground), dead organic matter, and soil carbon^61^. It also includes data on carbon remaining on harvested wood products, non-CO_2_ emissions, and foregone sequestration. The carbon stock data is combined with assumptions about carbon sequestration from forest growth (foregone if converted), mode of conversion, and CO_2_ emissions from land clearing using fire, and the fraction of carbon that remains sequestered in wood products during a 30-year time horizon. The AEZ-EF model is designed to estimate land use emissions from land use transitions predicted by comparative static economic models, whereby one starts with a baseline and estimates the resulting final equilibrium. The AEZ-EF model underlies the emission estimates in several analysis of the indirect land use effects of biofuels emissions and land conservation measures^15,62^.

## Supplementary information

Supplementary information

## Data Availability

The datasets generated during the current study are available in the Harvard Dataverse repository, 10.7910/DVN/DNF1WH.

## Code availability

The code to reproduce the results the current study is available in the Harvard Dataverse repository, 10.7910/DVN/DNF1WH.
