# Supplementary material for: Leakage does not fully offset soy supply-chain efforts to reduce deforestation in Brazil
Source: Nat Commun. 2022 Sep 17;13:5476. doi: 10.1038/s41467-022-33213-z (PMC9482629; doi:10.1038/s41467-022-33213-z)
Supplement: Supplementary file 1 — Supplementary information [file 41467_2022_33213_MOESM1_ESM.pdf]

Leakage does not fully offset soy supply-chain efforts to reduce deforestation in Brazil

Authors: Nelson Villoria, Rachael Garrett, Florian Gollnow, Kimberly Carlson

Supplementary Information

S1. Baseline Simulations and Model Validation

The base model is calibrated to the global equilibrium of production, consumption, and international trade in year 2011. The baseline simulation results from disturbing the base year equilibrium with a comprehensive set of economic shocks that capture the changes in the economy from 2011 to 2016. Therefore, the baseline simulation gives model estimates of how different the global economy in 2016 relative to is 2011. We closely follow the strategy of Yao et al. [1] and Taheripour et al. [2]. The baseline includes the macroeconomic shocks in table S1, the shocks to agricultural factor and input productivity in table S2 and the production shocks for oilseed in the BBAP region and China in table S3 (other region are shown for completeness). Shocks to the consumption of sugar cane, coarse grains, and vegetable oils for ethanol and biodiesel production are also included (14% and 56% increase in the demand of vegetable oils and coarse grains by the chemical sector in the EU, 19% increase in the demand of coarse grains for ethanol in the U.S., 10% increase in China, and 1% increase in the demand for raw sugar for ethanol in Brazil.)

Compared to observed exports, export predictions are highly accurate for Brazil, and to a lesser extent Bolivia (table S4). In the case of Argentina, the sharp contraction in the observed exports is due to producers hoarding soybean supplies during the 2016-2017 harvest as a response to delays in planned reduction of export taxes[3]. In the case of Paraguay—a very marginal direct soybean exporter to China—the rapid growth from a very low initial value is not captured by the model. Regarding the importance of the Chinese market, 38% of China's soybeans imports came from Brazil in 2011, and 14% from Argentina. Bolivia and Paraguay are marginal players. The main destination of Paraguayan oilseed is Brazil. Although trade supposedly liberalized in the region, it was not until 2016 that Argentina opened up its markets to Paraguayan soybeans in order to use underutilized soybean crushing capacity[3].

The comparisons of simulated and observed oilseed, cropland, and forest area in Fig. S3 are subject to some caveats. First, myriad drivers govern land use processes (e.g., market access, large scale fires, deforestation decisions responding to expectations of higher prices in the future, and land property rights), and our model is not designed to capture these drivers. Second, the translation of changes in land use calculated by applied equilibrium models rests on assumed land productivity which are highly uncertain (See section S3 for sensitivity analysis).

In addition to uncertainty in the inputs to the model, the comparisons to observed data should be taken as referential because there are important differences in the definitions of land cover used by FAOSTAT and the definitions used in GTAP. In particular, cropland in GTAP is equivalent to the sum of the harvested areas of all the crops in the model, while FAOSTAT includes all arable lands, meadows, and permanent crops. Regarding forests, the GTAP database only works with forests for which land rents can be imputed, which is a function of their accessibility [4]. This is a subset of all the forests in a country, which is what is reported in FAOSTAT. Finally, for

consistency, in GTAP, the weighted changes in land cover sum up to zero so changes in one land cover must be matched by changes in other land covers so that the total sum equals zero.

With these caveats in mind, there are still several important insights in figure S4. The model estimated change in Brazil's oilseed harvested area is a third of the value of the harvested area indicated by FAOSTAT. Because the simulated area is the area needed to produce the observed change in oilseed production (also from FAOSTAT, table S3), this suggests that the model is overestimating either the productivity of land or the input use response to changes in prices in Brazil. This is also true in Paraguay and Bolivia, but not in Argentina). Both responses, productivity, and input use, are regulated by model parameters (See sensitivity analysis in text S4).

In contrast, the model overestimates cropland expansion in Argentina and Brazil and underestimates cropland expansion in Bolivia and Paraguay. The drivers of cropland change include assumptions about productivity but also depend on demand for other land-based commodities and the supply response of all the regions in the model. Below we test the sensitivity of our results to different configurations of demand and productivity shocks.

In general, the model underestimates deforestation, which is not entirely surprising due to very different definitions of forests in GTAP (accessible forests for which a land rent can be imputed) which are a subset of the total forested area reported by FAOSTAT.

S2. Sensitivity analysis

<u>To Forest Definitions</u>

We allowed for two different definitions of forests in Brazil to accommodate different biome characteristics and ZDCP targets [5]. Definition A was exclusively based on mapped forest cover. Definition B included natural grasslands outside the Amazon Biome, which may have high conservation value and are included in some traders ZDCP definition [e.g., "Transforms our supply chain to be deforestation free while protecting native vegetation beyond forests." [6]]. Our main results are robust to the two different definition of forests we employ in the paper (Fig. S4).

<u>To Model Parameters</u>

To bound the uncertainty of results due to uncertainty in model parameters we conduct systematic sensitivity analysis of the deforestation outcomes in the BBAP region to uncertainty in a set of key parameters [7,8]. Systematic sensitivity analysis consists in solving the model repeatedly, each time under a different set of parameter values that span the range of the plausible distribution of the underlying unobserved *true* parameter. We use the Gaussian quadrature techniques customary in the applied general equilibrium literature [9,10]. Gaussian quadrature sampling allows for a drastic reduction of the samples needed to obtain meaningful estimates of the mean and standard deviation of modeling outcomes conditional on specific parameter values. We follow closely Hertel et al. [7], and focus on the following parameters:

- The elasticity of crop yields with respect to crop prices, which regulates the ease with which land can be substituted for other inputs to increase yields. The default GTAP-AEZ value is 0.25, and we allow it to vary between 0 and 0.5, assuming a triangular distribution.

- The elasticities of transformation between forests and agricultural lands and between cropland and pastures (Ω_1 and Ω_2 in Fig. S4). We vary these two parameters by 80% assuming the values follow a triangular distribution.

- The elasticity of substitution of imports across different exporters (Armington Elasticities). The means and standard deviations of these parameters were estimated by Hertel et al. [11].

- The elasticity of effective crop land with respect to harvested crop land expansion. The GTAP-AEZ model measures land in units of output value, or effective land. An effective unit of land combines both the physical area of land as well as its yield [12]. An increment in effective cropland translates into an increase in physical cropland that needs to be adjusted by the yield of the land in which cropland is expanding. In the absence of published values of these elasticities [7], the standard GTAP-AEZ model assumes that marginal lands brought into crop production are two-thirds as productive as lands currently in use, which is captured by the elasticity of effective cropland (ETA) with respect to harvested crop land expansion (ETA=0.66). In other words, a hectare of current land would need to be replaced for three hectares of marginal lands to get the same output. This value in line with the assumption that marginal lands are much less productive than the average of land currently in production. Recent estimates using terrestrial ecosystem models (TEM) suggest that the productivity of marginal lands in Brazil and other tropical countries is close to those of land under production (ETA $\approx$ 0.9) [13]; in contrast, evidence from stochastic frontier analysis, suggest a much larger gap between the natural productivity of land in the Cerrado relative to established farms elsewhere in Brazil; available estimates indicate that farms in Cerrado use 2.8 more (quality-adjusted) land than farms elsewhere [14]—this is a result consistent with ETA=0.66. Given this evidence, we set the default value of this parameter as 0.66 and we allow it to vary symmetrically (using a triangular distribution) by an ordinary change of 0.24, which effectively imposes an upper bound of 0.90, the maximum value reported in the literature [13].

The confidence intervals estimated for the forest outcomes in Brazil indicate that such outcomes are statistically significant in the sense that zero is not contained in the intervals (Fig. S5. For Argentina, Bolivia and Paraguay, the confidence intervals contain zero, indicating that parameter uncertainty is more important for these regions. These results underscore the urgency of refining land use parameters for agricultural frontiers countries of South America outside of Brazil.

As an additional sensitivity test, Fig. S6 contrasts our estimates of net avoided deforestation in Brazil using the default ETA value of 0.66 as well as the estimates from terrestrial ecosystem models [13], which are close to 0.9. The default values tend to overestimate net avoided deforestation relative to the TEM estimates by approximately 8% (i.e., 25 kha in the case of the ASM, 42 kha in the global ZDCP scenario, and just above 50 kha in the other three scenarios.)

S3. Further Details on Comparison with Existing Estimates

Two recent articles report estimates of avoided deforestation in Brazil due to ZDCPs in soy supply chains in Brazil. Heilmayr et al. [15] use a triple difference-in-difference regression design to isolate the effect of the ASM using fine scale data of the Amazon Biome during the period

2006-2016. Their regression counterfactual indicates that the ASM saved an estimated 1,800 kha +/- 900 kha, with a 95% confidence interval of 900-2,700 kha. On an annualized basis this implies land savings of 90-270 kha/year. Our estimate under the most stringent market share threshold of ≥75% indicates avoided deforestation in the Amazon of 409 kha during 2011-2016, or 82 kha/year. Our estimates are therefore closest to the lower bound estimate reported in Heilmayr et al. [15]. This is not surprising since regression counterfactual approaches ignore spillover and feedback effects via trade, commodity, and factor markets, as well as adjustments in utilization by both producers and consumers as a response to changes in prices, and therefore tend to overestimate land savings from conservation programs[16].

Soterroni et al.[17] use a partial equilibrium model focused on Brazil to simulate the effects of extending the ASM to the Cerrado. They estimate reductions in the conversion of native vegetation (relative to a baseline without restrictions in Cerrado) of 3,600 kha during 2020-2050. This translates into avoided deforestation of 120 kha per year. In our study, the scenario that most closely resembles an extension of the ASM to Cerrado is the scenario "Global Zero Deforestation Commitments (incl. ASM)" (Figs. 1c, 1g, 2b). Under this scenario, we estimate a net avoided deforestation during 2011-2016 of 38.3 kha in the most restrictive scenario (≥75% market share threshold, Fig. 2b), or 7.6 kha/year. To some extent, the difference between our results may be related to the fact that we only extend the ASM to municipalities in the Cerrado (and other biomes) that have committed companies with market shares large enough to assume compliance with land conversion restrictions. More fundamentally, the differences in modeling strategies and time horizons of the two studies preclude a proper comparison. For, instance, as indicated in Fig. S1, cumulative deforestation for soy in the Amazon amounted to 117 kha. At an estimated rate of 120 kha/year, Soterroni et al's[17] results would have implied large reforestation in the Cerrado over 2011-2016. In contrast, our estimate implies that extending the ASM to Cerrado during 2011-2016 would have reduced observed deforestation by 33%.

S4. United States Supply Response

The ASM reduced soybean production in Brazil by 40.7 kilotonnes from 2011 to 2016 (using the 75% market share threshold (Fig. S7). Brazil's production in 2016 was of 98,153 kt [18], so the reduction is merely 0.04%. Most of the reduction in Brazil's production is offset by an increase in soybean and other oilseed production in the rest of the world, particularly in North America and in the EU (Fig. S8). In both regions, the expansion of soybeans and other oilseeds comes mostly from crop reallocation, with almost negligible cropland expansion, even in the least restrictive market share thresholds (figs. S9, S10).

Supplementary Figures (extended Data upon publication)

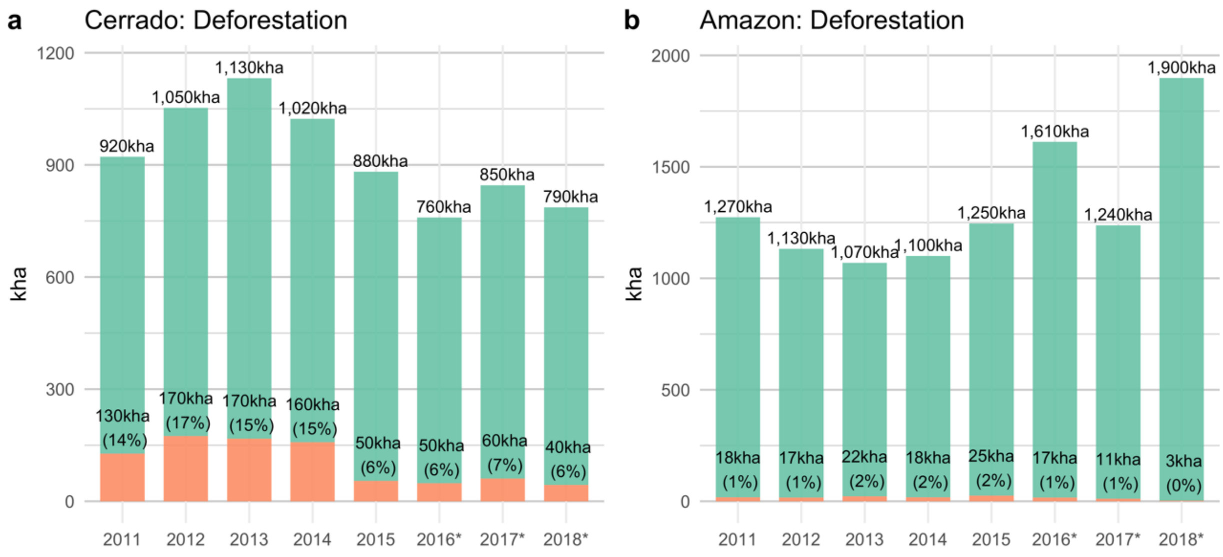

Fig. S1. Deforestation and five-year soy-deforestation as derived from Mapbiomas[19] **for the Brazilian Amazon (a) and Cerrado (b).** * Indicates years with an underestimation of soy-deforestation, due to limited post-deforestation observation years.

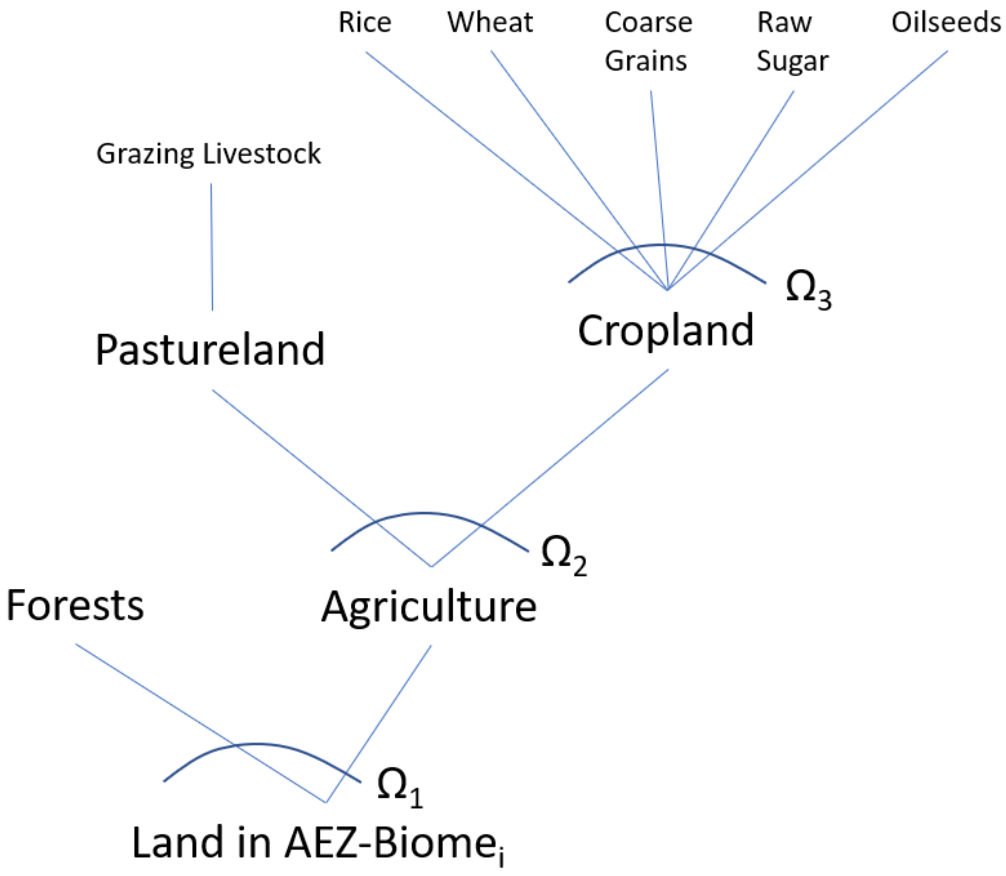

Fig. S2. Land Supply Structure in the GTAP AEZ Model with Biome Splits. At the top of the Constant Elasticity of Transformation (CET) nesting structure, a representative profit-maximizing producer operating under constant returns to scale chooses the crop mix that maximizes profits subject to resource and technological constrains. The parameter _3 is an elasticity of transformation that captures the ease of converting cropland into each use. The mix of crops generates a change to the rents accruing to cropland. The optimal allocation between cropland and pastureland is determined by comparing the changes in the rents accruing to the two uses; _2 captures the ease with which pasturelands can be converted to croplands, and vice versa. At the bottom of the supply tree, the land rents for the overall agricultural activity are compared to the rents earned by forested lands. The relative size of these rents in conjunction with the elasticity of transformation Ω_1 determine the ease with which forests are converted into cropland, and vice versa. We implement Zero Deforestation Commitments by setting _1 equal to zero for the intersections of Agroecological Zones (AEZ) and Biomes (Cerrado and Amazonia) where companies with ZDCPs have a dominant market share.

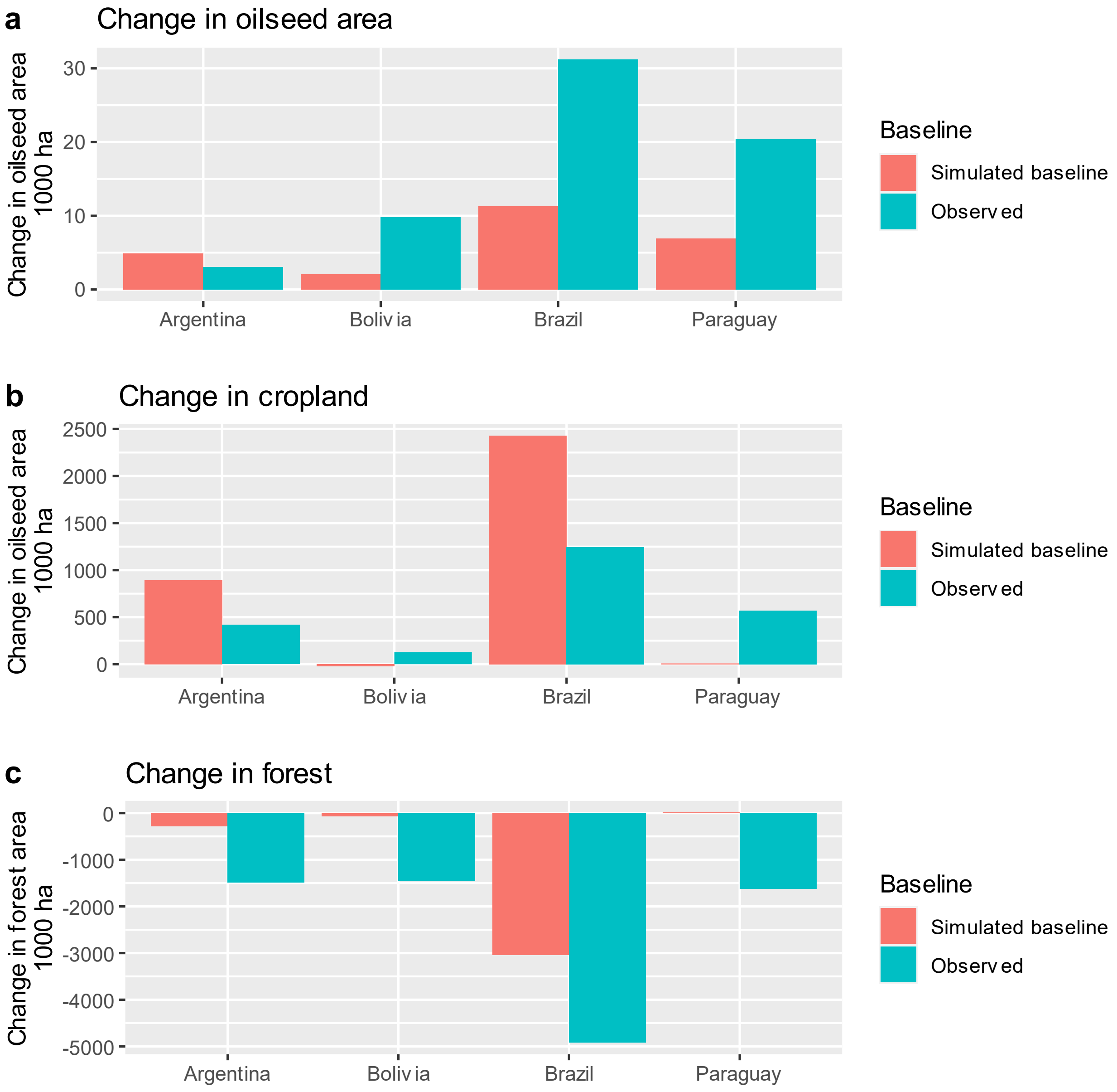

Fig. S3. Simulated baseline and observed changes in oilseed (a), cropland (b), and forest (c) area from 2011 to 2016. The model correctly predicts the direction of changes in oilseed land use and cropland and forest covers across the BBAP region.

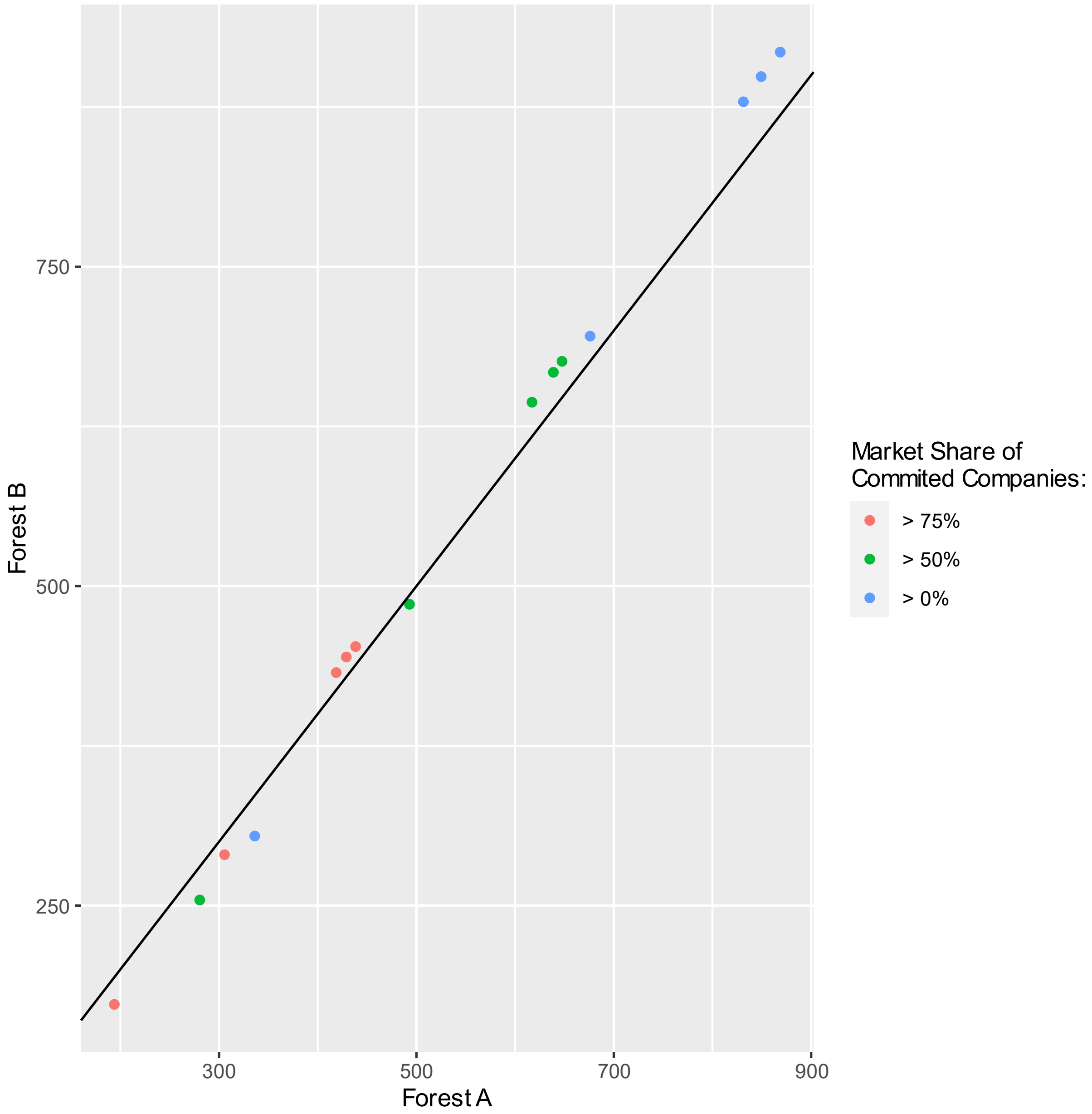

Fig. S4. Net avoided deforestation in Brazil by forest type and market share threshold for compliance. In forest definition "A" forests are defined as forest only, as mapped by PRODES for the Amazon and by Mapbiomas for other biomes [20]. In forest definition "B" forest are defined as forest only in the Amazon biome, as mapped by PRODES, and forest and grasslands in all other biomes, as mapped by Mapbiomas. Values close to the black line indicate similar results. Market shares indicate the threshold (% market share) at which producers within a municipality comply with zero-deforestation requirements (i.e., no deforestation after 2011). Results are qualitatively similar across forest definitions.

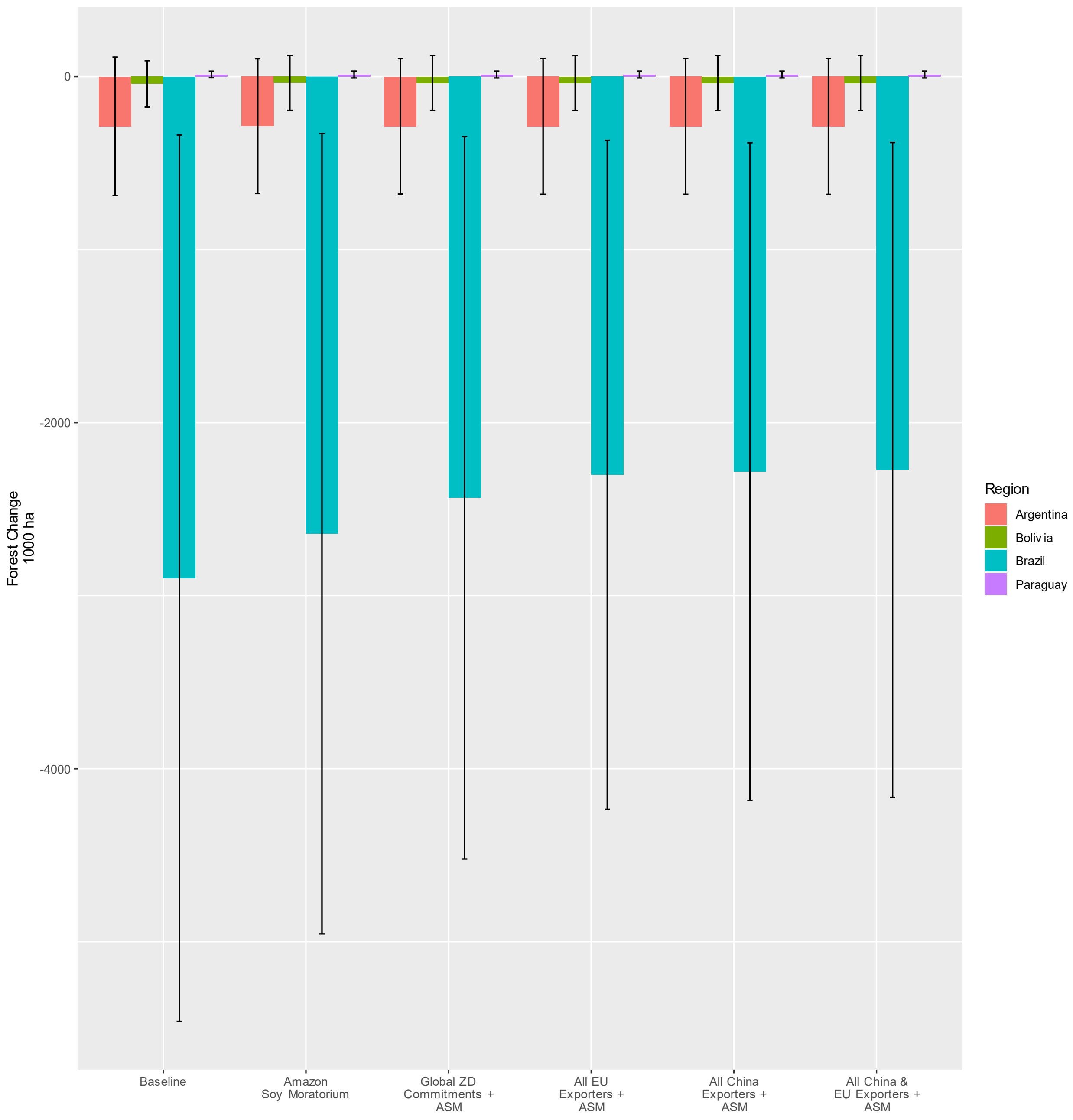

Fig. S5. Forest Change (2011-2016) in ZDCP Scenarios compared to baseline in Brazil, Bolivia, Argentina, and Paraguay. Error bars extend two standard deviations and represent the uncertainty in model results due to uncertainties in crop yield price elasticities, elasticities of transformation between forests and agricultural lands and between cropland and pastures, trade elasticities, and the elasticity of effective cropland with respect to harvested cropland expansion. These are 95% confidence intervals under normality and 75% Chebyshev confidence intervals for unknown distributions. Results are for market share threshold> 75% (companies with ZDCP commitments have at least a 75% export market share.)

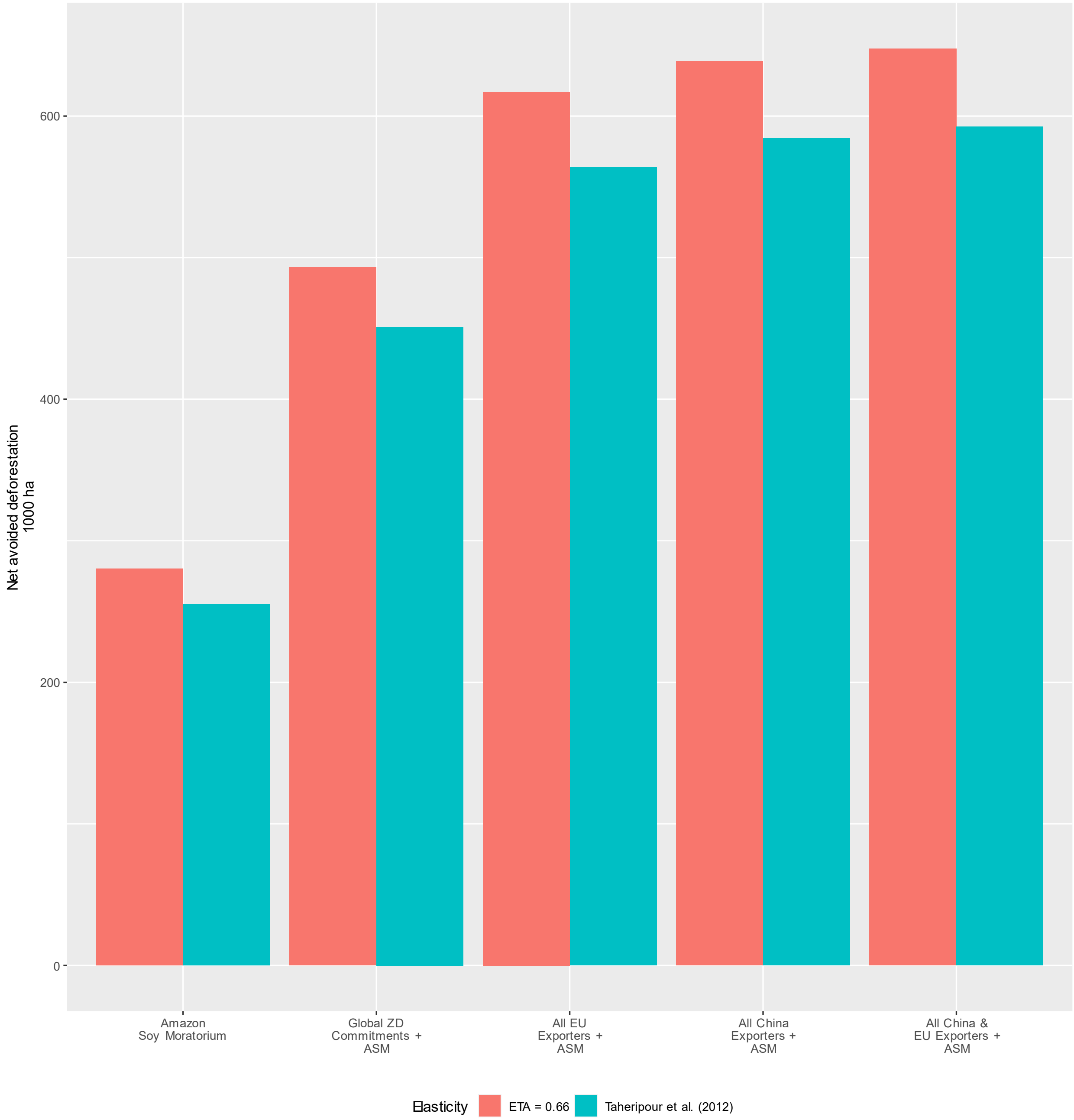

Fig. S6. Net avoided deforestation under different assumptions about the value of the elasticity of effective crop land with respect to harvested crop land expansion (ETA). The values reported by Taheripour et al [13] are close to unity.

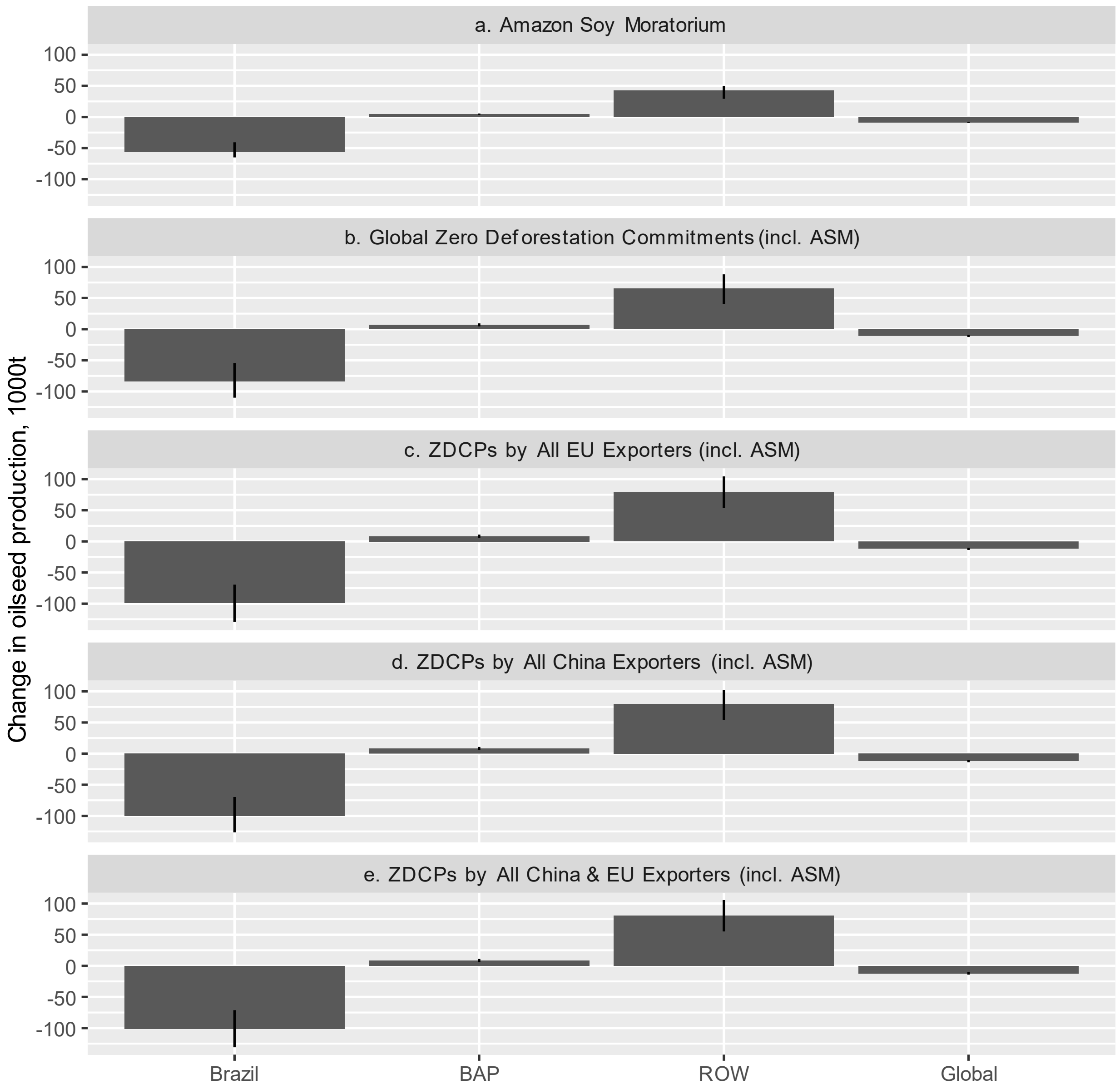

Fig. S7 Changes in Oilseed Production Under Different Zero Deforestation Commitment scenarios, by region. a: Amazon Soy Moratorium. **b:** Global Zero Deforestation Commitments. **c:** All EU Exporters. **d:** All China Exporters. **e:** All China & EU Exporters. **b-e** include the Amazon Soy Moratorium. BAP = Brazil, Argentina, Paraguay; ROW = Rest of World; Global = net global effect (Brazil + BAP + ROW). Vertical lines provide the range of outcomes based on >0% [top of line], ≥50% [intersection of line with bar] and ≥75% [bottom of line] thresholds used to determine if a municipality is subject to zero-deforestation supply chain policies.

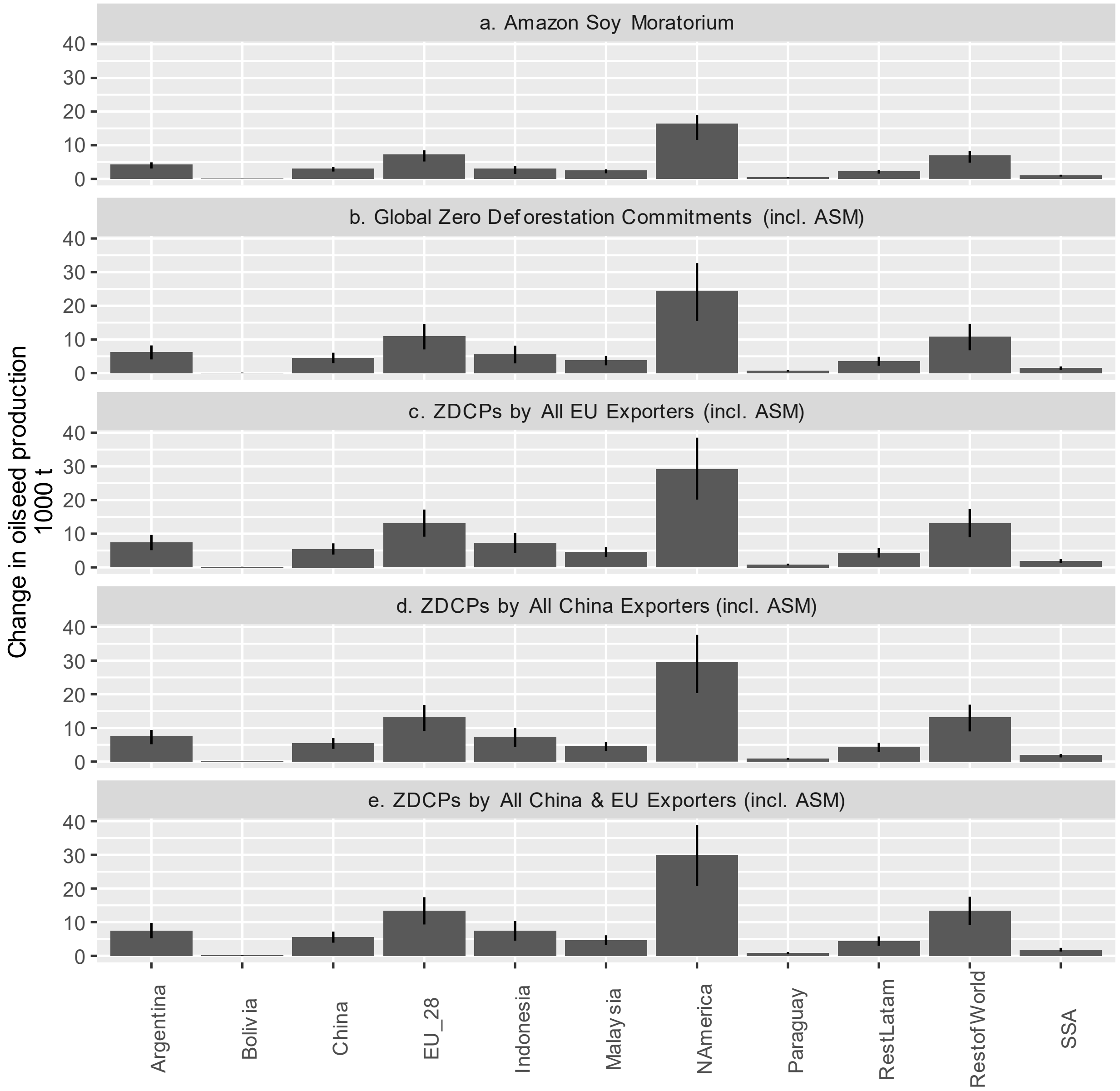

Fig. S8. Changes in Oilseed Production Under Different Zero Deforestation Commitment scenarios, by country. a: Amazon Soy Moratorium. **b:** Global Zero Deforestation Commitments. **c:** All EU Exporters. **d:** All China Exporters. **e:** All China & EU Exporters. **b-e** include the Amazon Soy Moratorium. SSA = sub-Saharan Africa. Vertical lines provide the range of outcomes based on >0% [top of line], ≥50% [intersection of line with bar] and ≥75% [bottom of line] thresholds used to determine if a municipality is subject to zero-deforestation supply chain policies.

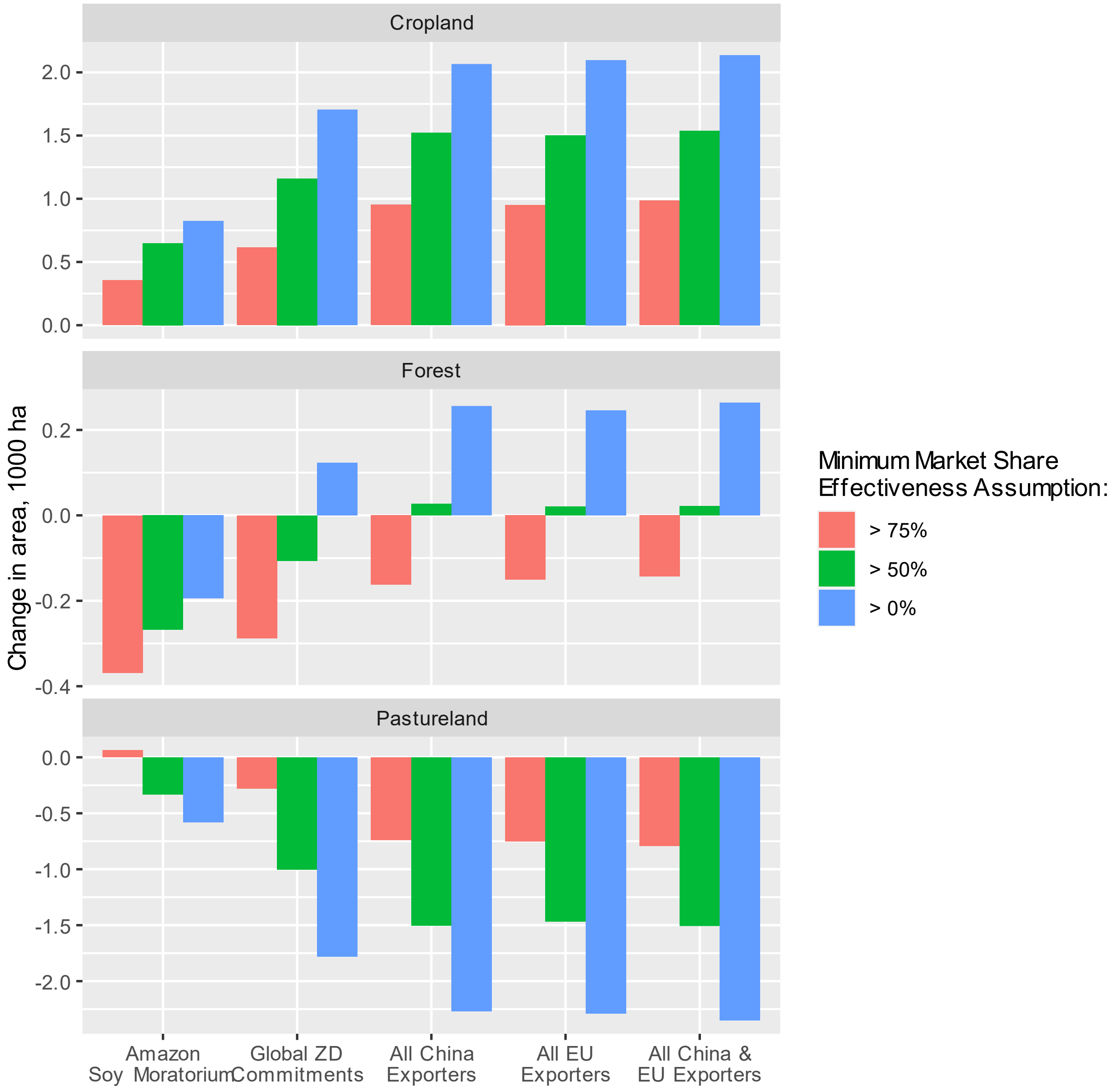

Fig. S9. Land Cover Changes in North America Under Different Zero Deforestation Corporate Policy scenarios. Market shares indicate the threshold (% market share) at which producers within a municipality comply with zero-deforestation requirements (i.e., no deforestation after 2011).

Tables

Table S1: Percentage Change in Macroeconomic Drivers of Land Use Between 2011 and 2016

Region	Capital Stocks	Employment	Investments	Population	Real GDP
Argentina	22.72	4.58	-7.39	5.11	3.23
Bolivia	40.38	4.62	33.56	7.73	25.78
Brazil	23.14	5.10	-34.88	4.41	0.62
China	59.74	1.59	25.10	2.59	36.10
EU_28	22.44	2.17	6.96	0.68	6.84
Indonesia	43.16	8.01	22.60	6.07	25.84
Malaysia	35.86	15.05	21.07	8.49	25.41
North America	14.92	6.65	8.34	3.67	10.74
Paraguay	15.95	9.61	22.85	6.62	21.37
Rest Latin America	28.75	10.13	0.74	6.39	-1.87
Rest of the World	NA	NA	7.49	6.56	15.41
SSA	35.92	16.63	16.22	13.06	18.87

Notes: For all the variables we take the averages during 2010-2012 and 2015-2017 as endpoints of the period. Sources: Real GDP (Output side) comes from the World Development Indicators [21]. The rest of the variables come from the Penn World Tables 9.1 [22].

Table S2 Percentage Change in Agricultural Input and Factor Productivity between 2011 and 2016

Region	Capital Productivity	Fertilizer Productivity	Labor Productivity
Argentina	10.07	7.51	12.86
Bolivia	6.33	-22.06	7.96
Brazil	0.49	-6.97	34.36
China	-16.29	8.47	44.09
EU_28	7.07	4.92	23.56
Indonesia	-16.17	-8.91	18.13
Malaysia	-5.73	-5.76	20.80
North America	9.23	-2.36	17.22
Paraguay	15.90	-10.44	20.42
Rest of Latin America	9.16	-0.61	14.69
Rest of the World	1.37	-2.43	16.48
SSA	3.92	-9.11	6.74

Notes: Productivity is defined as 2004-2006 international US dollars per 40 CV tractor-equivalent machinery unit (proxy for capital); per metric ton of N, P205, and K2 nutrients of fertilizer consumed (fertilizer); per 1000 persons economically active in agriculture, +15 yrs, male+female. We estimate the growth rate of each ratio during 2009-2015 (last available year) and then calculate the net percentage change between 2016 and 2011. Source: International Productivity Dataset, USDA Economic Research Service which is based on FAOSTAT [23].

Table S3. Percentage Change in Harvested Area and Production of Oilseed Between 2011 and 2016

GTAP Sector	Region	Area	Production
Oilseeds	Argentina	3.07	21.24
Oilseeds	Bolivia	9.79	40.60
Oilseeds	Brazil	31.24	38.37
Oilseeds	China	-11.15	-3.30
Oilseeds	EU_28	4.73	13.07
Oilseeds	Indonesia	33.58	38.16
Oilseeds	Malaysia	0.62	5.57
Oilseeds	North America	11.97	30.28
Oilseeds	Paraguay	20.37	33.64
Oilseeds	Rest of Latin America	33.78	28.04
Oilseeds	Rest of the World	10.88	10.76
Oilseeds	SSA	8.57	11.99

Notes: For all the variables we take the averages during 2010-2012 and 2015-2017 as endpoints of the period. Sources: FAOSTAT [18].

Table S4. Observed and simulated percent changes in oilseed exports from BBAP (Brazil, Bolivia, Argentina, Paraguay) to China, from 2011 to 2016, using baseline shocks.

Region	Simulated Baseline	Observed
Argentina	29.69	-27.15
Bolivia	131.95	138.54
Brazil	52.83	69.56
Paraguay	56.79	1097.28

References

1. Yao, G., Hertel, T. W. & Taheripour, F. Economic drivers of telecoupling and terrestrial carbon fluxes in the global soybean complex. *Global Environmental Change* **50**, 190–200 (2018).
2. Taheripour, F., Hertel, T. W. & Ramankutty, N. Market-mediated responses confound policies to limit deforestation from oil palm expansion in Malaysia and Indonesia. *PNAS* 201903476 (2019) doi:10.1073/pnas.1903476116.
3. Mergen, D. & Sandoval, L. 2017/18 Forecast: Limited area growth for Soybeans, Sunflower, and Peanuts. 14 (2017).
4. Sohngen, B., Tennity, C., Hnytka, M. & Meeusen, K. Global Forestry Data for the Economic Modeling of Land Use. in *Economic Analysis of Land Use in Global Climate Change Policy* (eds. Hertel, T. W., Rose, S. K. & Tol, R. S. J.) 49–72 (Center for Global Trade Analysis, Department of Agricultural Economics, Purdue University, 2009).
5. INPE. *Deforestation – Legal Amazon*. http://terrabrasilis.dpi.inpe.br/downloads/.
6. CARGIL. *Cargill Policy on Sustainable Soy – South American Origins*. https://www.cargill.com/doc/1432136544508/cargill-policy-on-south-american-soy.pdf (2019).
7. Hertel, T. W. *et al.* Effects of US Maize Ethanol on Global Land Use and Greenhouse Gas Emissions: Estimating Market-Mediated Responses. *BioScience* **60**, 223–231 (2010).
8. Lobell, D. B., Baldos, U. L. C. & Hertel, T. W. Climate Adaptation as Mitigation: The Case of Agricultural Investments. *Environ. Res. Lett.* **8**, 015012 (2013).
9. DeVuyst, E. A. & Preckel, P. V. Sensitivity analysis revisited: A quadrature-based approach. *Journal of Policy Modeling* **19**, 175–185 (1997).

10. Pearson, K. & Arndt, C. *Implementing Systematic Sensitivity Analysis Using GEMPACK*. (2000).

11. Hertel, T. W., Hummels, D., Ivanic, M. & Keeney, R. How confident can we be of CGE-based assessments of Free Trade Agreements? *Economic Modelling* **24**, 611–635 (2007).

12. Hertel, T. W., Lee, H.-L., Rose, S. & Sohngen, B. Modeling Land-Use Related Greenhouse Gas Sources and Sinks and Their Mitigation Potential. in *Economic Analysis of Land Use in Global Climate Change Policy* (eds. Hertel, T. W., Rose, S. & Tol, R.) 123–154 (Routledge, 2009).

13. Taheripour, F., Zhuang, Q., Tyner, W. E. & Lu, X. Biofuels, cropland expansion, and the extensive margin. *Energy, Sustainability and Society* **2**, 25 (2012).

14. Rada, N. Assessing Brazil's Cerrado agricultural miracle. *Food Policy* **38**, 146–155 (2013).

15. Heilmayr, R., Rausch, L. L., Munger, J. & Gibbs, H. K. Brazil's Amazon Soy Moratorium reduced deforestation. *Nature Food* **1**, 801–810 (2020).

16. Hertel, T. W. Economic perspectives on land use change and leakage. *Environ. Res. Lett.* **13**, 075012 (2018).

17. Soterroni, A. C. *et al.* Expanding the Soy Moratorium to Brazil's Cerrado. *Science Advances* **5**, eaav7336 (2019).

18. FAO. FAOSTAT. *Food and Agriculture Organization of the United Nations Statitstical Database* http://faostat.fao.org/. Accessed: 07-15-2017 (2021).

19. MapBiomas. Project MapBiomas - Collection v5.0 of Brazilian Land Cover & Use Map Series. http://mapbiomas.org/ (2020).

20. INPE. Monitoramento da Cobertura Florestal da Amazônia por Satélites: Sistemas Prodes. (2018).

21. WDI. World Bank's World Development Indicators Online. http://go.worldbank.org/6HAYAHG8H0. Accessed: 12/14/2017 (2020).

22. Feenstra, R. C., Inklaar, R. & Timmer, M. P. The Next Generation of the Penn World Table. *American Economic Review* **105**, 3150–3182 (2015).

23. Fuglie, K. O. USDA ERS - International Agricultural Productivity. https://www.ers.usda.gov/data-products/international-agricultural-productivity. Accessed: 02-28-2017 (2017).
